# Supplementary material for: Evaluation of the Effects of Heteroaryl Ethylene Molecules in Combination with Antibiotics: A Preliminary Study on Control Strains
Source: Antibiotics (Basel). 2023 Aug 10;12(8):1308. doi: 10.3390/antibiotics12081308 (PMC10451629; doi:10.3390/antibiotics12081308)
Supplement: Supplementary file 1 [file antibiotics-12-01308-s001.zip › antibiotics-2542410-supplementary.pdf]

Supplementary Materials

Evaluation of Synergistic Effect of Heteroaryl Ethylene Molecules in Combination with Antibiotics: A Preliminary Study on Control Strains

Carmelo Bonomo [1,†], Paolo Giuseppe Bonacci [1,†], Dalida Angela Bivona [1], Alessia Mirabile[1], Dafne Bongiorno [1,*], Emanuele Nicitra [1], Andrea Marino [2], Carmela Bonaccorso [3,*], Giuseppe Consiglio [3], Cosimo Gianluca Fortuna [3], Stefania Stefani [1] and Nicolò Musso [1]

[1] Department of Biomedical and Biotechnological Sciences (BIOMETEC), Università degli Studi di Catania, Via S. Sofia, 89, 95123 Catania, Italy. carmelo.bonomo@phd.unict.it (C.B.); paolo.bonacci@phd.unict.it (P.G.B.); dalida.bivona@phd.unict.it (D.A.B.); alessiamirabile93@gmail.com (A.M.); dbongio@unict.it (D.B.); emanuelenicitra@gmail.com (E.N.); stefanis@unict.it (S.S.); nmusso@unict.it (N.M.).

[2] Unit of Infectious Diseases, Department of Clinical and Experimental Medicine, ARNAS Garibaldi Hospital, Università degli Studi di Catania, Via Palermo, 95122 Catania, Italy. andreamarino9103@gmail.com (A. Ma.).

[3] Department of Chemical Sciences, Università degli Studi di Catania, Viale Andrea Doria 6, 95125 Catania, Italy. carmela.bonaccorso@unict.it (C. Bona.); giuseppe.consiglio@unict.it (G.C.); cg.fortuna@unict.it (C.G.F.).

* Correspondence: dbongio@unict.it (Microbiology Section), +393921210540; carmela.bonaccorso@unict.it (Chemistry Section), +393284723158-

† These authors contributed equally

Table of contents

Table S1. Structures of the 13 compounds added to the QSAR model for antimicrobial activity against *S. aureus* ATCC29213 ***S2***

Figure S1. Score plot of Principal Component Analysis (PCA) at the third component (PC1 *vs* PC2 *vs* PC3) for the 49 heteroaromatic compounds tested for antimicrobial activity against *S. aureus* ATCC29213. ***S4***

Figure S2. Plot of the coefficient of determination R^2 and cross-validated coefficient of determination (leave-one-out, LOO) Q^2 vs. the number of LVs of the PLS models for antimicrobial activity against *S. aureus* ATCC29213. ***S4***

Figure S3. Plot of the Variable Influence on Projection VIP of the PLS models for antimicrobial activity against *S. aureus* ATCC29213. ***S5***

Figure S4. Plot of the Weights for VS+ descriptors at the third latent variable (LV1 *vs* LV2 *vs* LV3) of the PLS models for antimicrobial activity against *S. aureus* ATCC29213. ***S5***

Table S2. Structures of the 38 heteroaryl ethylene compounds of the QSAR model for cytotoxic activity towards CaCo2 colon-rectal cancer cell line ***S6***

Figure S5. Score plot of Principal Component Analysis (PCA) at the third component (PC1 *vs* PC2 *vs* PC3) for the 38 Heteroaromatic compounds tested for cytotoxic activity towards CaCo2 colon-rectal cancer cell line. ***S12***

Figure S6. Plot of the coefficient of determination R^2 and cross-validated coefficient of determination (leave-one-out, LOO) Q^2 vs. the number of LVs of the PLS models for cytotoxic activity towards CaCo-2 colon-rectal cancer cell line. ***S12***

Figure S7. Plot of the Variable Influence on Projection VIP of the PLS models for cytotoxic activity towards CaCo-2 colon-rectal cancer cell line. ***S13***

Figure S8. Plot of the Weights for VS+ descriptors at the third latent variable (LV1 *vs* LV2 *vs* LV3) of the PLS models for cytotoxic activity towards CaCo-2 colon-rectal cancer cell line. ***S13***

Table S3. Dunnett's multiple comparisons test performed for all combinations. ***S14***

Table S1. Structures of the 13 compounds added to the QSAR model for antimicrobial activity against *S. aureus* ATCC29213 [9].

Molecules & Smiles string	ID VS+	MIC (µg/mL)	[Ref]
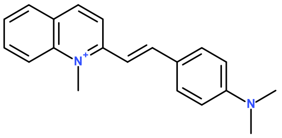<br>CN(C)c1ccc(\C=C\c2ccc3ccccc3[n+]2C)cc1	**PB1**	4	
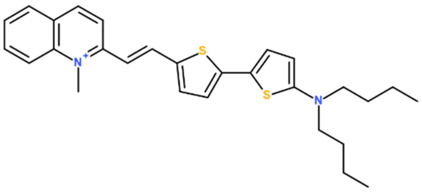<br>CCCCN(CCCC)c1ccc(s1)c2ccc(\C=C\c3ccc4ccccc4[n+]3C)s2	**PB2**	4	
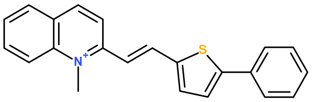<br>C[n+]1c(\C=C\c2ccc(s2)c3ccccc3)ccc4ccccc14	**PB3**	4	
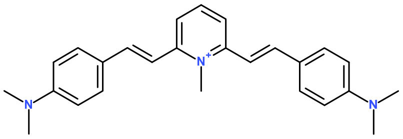<br>CN(C)c1ccc(\C=C\c2cccc(\C=C\c3ccc(cc3)N(C)C)[n+]2C)cc1	**PB4**	0.25	[9]
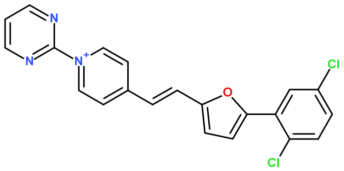<br>Clc1ccc(Cl)c(c1)c2oc(\C=C\c3cc[n+](cc3)c4ncccn4)cc2	**PB5**	1	
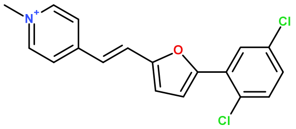<br>C[n+]1ccc(\C=C\c2oc(cc2)c3cc(Cl)ccc3Cl)cc1	**PB6**	4	
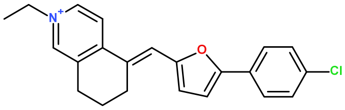<br>CC[n+]1ccc2\C(=C\c3oc(cc3)c4ccc(Cl)cc4)\CCCc2c1	**PB7**	4	

Table S1. Continued

<table>
<tr><td>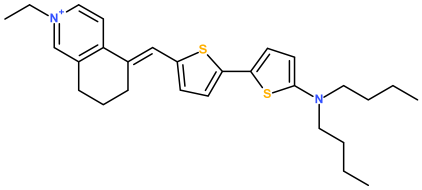
CCCCN(CCCC)c1ccc(s1)c2ccc(\C=C\3/CCCc4c[n+](CC)ccc34)s2</td><td>PB8</td><td>2</td><td>[9]</td></tr>
<tr><td>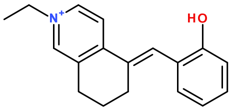
CC[n+]1ccc2\C(=C\c3ccccc3O)\CCCc2c1</td><td>BCNAc1</td><td>>128</td><td rowspan="3">Unpublished results</td></tr>
<tr><td>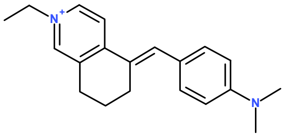
CC[n+]1ccc2\C(=C\c3ccc(cc3)N(C)C)\CCCc2c1</td><td>BCM3</td><td>64</td></tr>
<tr><td>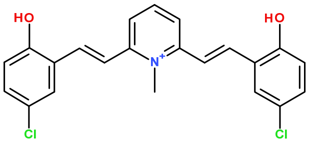
C[n+]1c(\C=C\c2cc(Cl)ccc2O)cccc1\C=C\c3cc(Cl)ccc3O</td><td>BCM12</td><td>>128</td></tr>
<tr><td>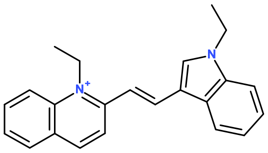
CC[n+]1c(\C=C\c2cn(CC)c3ccccc23)ccc4ccccc14</td><td>SQL</td><td>4</td><td rowspan="2">[22]</td></tr>
<tr><td>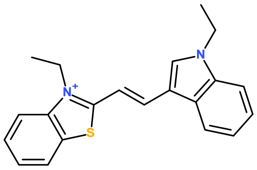
CC[n+]1c(\C=C\c2cn(CC)c3ccccc23)sc4ccccc14</td><td>SBT</td><td>1</td></tr>
</table>

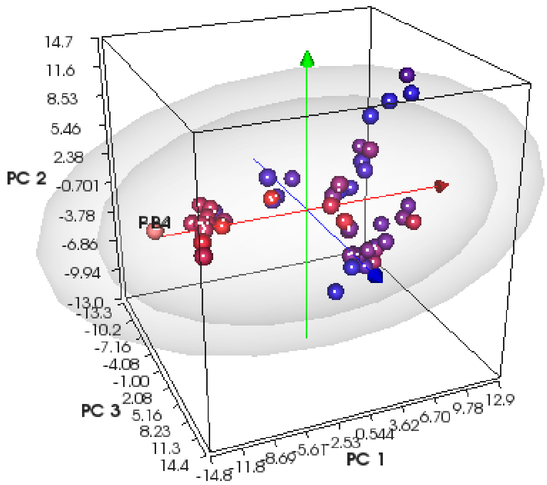

Figure S1. Score plot of Principal Component Analysis (PCA) at the third component (PC1 *vs* PC2 *vs* PC3) for the 49 Heteroaromatic compounds tested for antimicrobial activity against *S. aureus* ATCC29213. Compounds are color-coded by their activity values, using a scale from red (actives) to blue (inactives), according to the experimental MIC values.

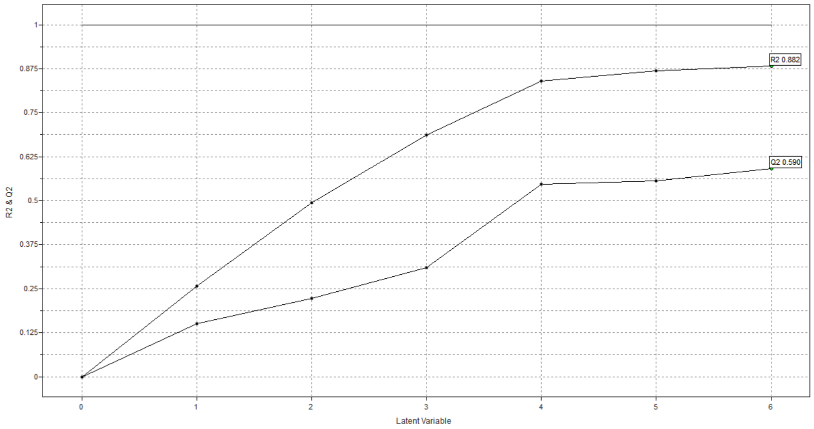

Figure S2. Plot of the coefficient of determination R^2 and cross-validated coefficient of determination (leave-one-out, LOO) Q^2 vs. the number of LVs of the PLS models for antimicrobial activity against *S. aureus* ATCC29213.

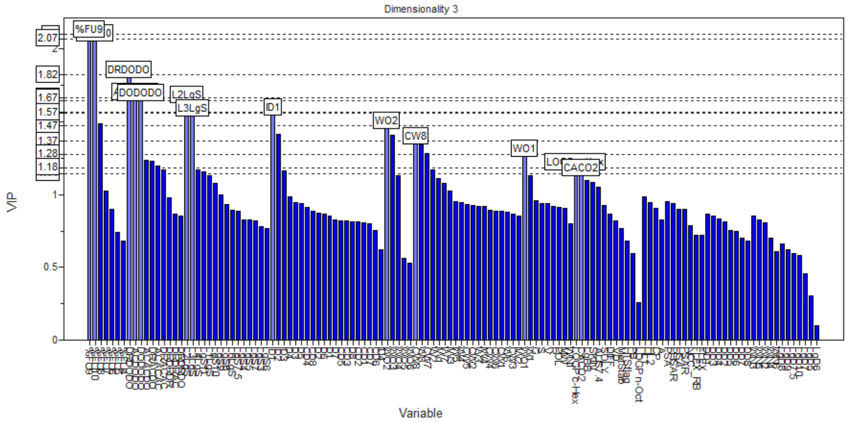

Figure S3. Plot of the Variable Influence on Projection VIP of the PLS models for antimicrobial activity against *S. aureus* ATCC29213.

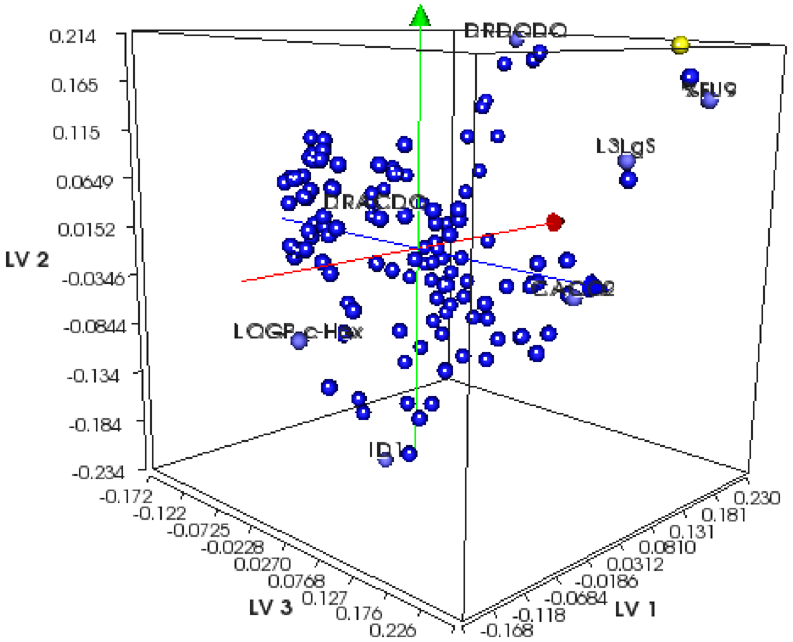

Figure S4. Plot of the Weights for VS+ descriptors at the third latent variable (LV1 *vs* LV2 *vs* LV3) of the PLS models for antimicrobial activity against *S. aureus* ATCC29213. The yellow circle represents the dependent variable (MIC).

Table S2. Structures of the 38 heteroaryl ethylene compounds of the QSAR model for cytotoxic activity towards CaCo2 colon-rectal cancer cell line

**Molecules & Smiles string**	**ID VS+**	**IC_{50} 48h**	**$Log(IC_{50})$ 48h**
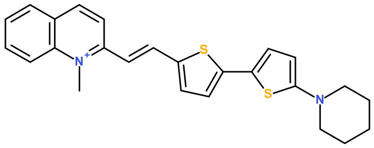 C[n+]1c(\C=C\c2ccc(s2)c3ccc(s3)N4CCCCC4)ccc5ccccc15	BC1	20	1.30
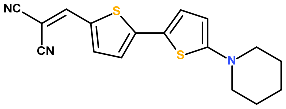 N#CC(=Cc1ccc(s1)c2ccc(s2)N3CCCCC3)C#N	BC2	100	2
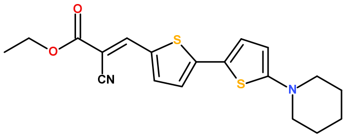 CCOC(=O)\C(=C\c1ccc(s1)c2ccc(s2)N3CCCCC3)\C#N	BC3	250	2.40
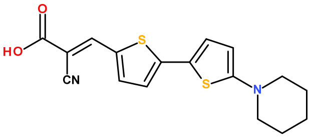 OC(=O)\C(=C\c1ccc(s1)c2ccc(s2)N3CCCCC3)\C#N	BC4	250	2.40
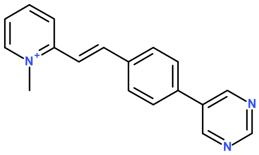 C[n+]1ccccc1\C=C\c2ccc(cc2)c3cncnc3	BC5	100	2
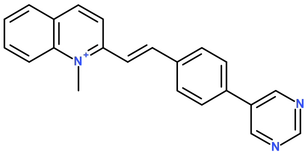 C[n+]1c(\C=C\c2ccc(cc2)c3cncnc3)ccc4ccccc14	BC6	25	1.5

Table S2. Continued

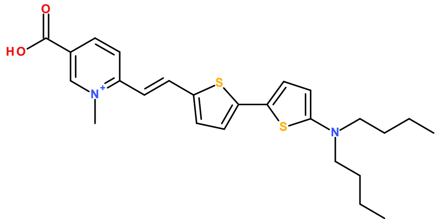 CCCCN(CCCC)c1ccc(s1)c2ccc(\C=C\c3ccc(c[n+]3C)C(=O)O)s2	BCG3	8	0.90
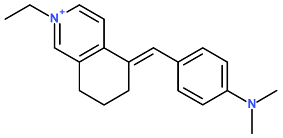 CC[n+]1ccc2\C(=C\c3ccc(cc3)N(C)C)\CCCc2c1	BCM3	1.35	0.13
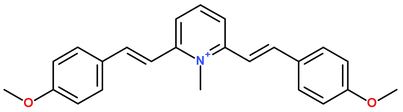 COc1ccc(\C=C\c2cccc(\C=C\c3ccc(OC)cc3)[n+]2C)cc1	BCM6	12.34	1.09
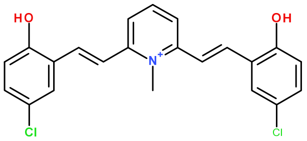 C[n+]1c(\C=C\c2cc(Cl)ccc2O)cccc1\C=C\c3cc(Cl)ccc3O	BCM12	250	2.40
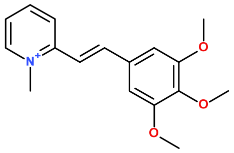 COc1cc(\C=C\c2cccc[n+]2C)cc(OC)c1OC	GC VI 2	250	2.40
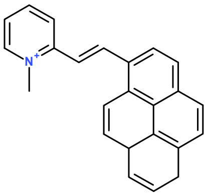 C[n+]1ccccc1\C=C\c2ccc3ccc4CC=CC5C=Cc2c3c45	GC VI 3	0.9	-0.0458
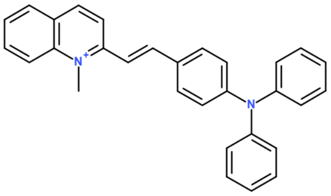 C[n+]1c(\C=C\c2ccc(cc2)N(c3ccccc3)c4ccccc4)ccc5ccccc15	GC VI 14	4	0.602

Table S2. Continued

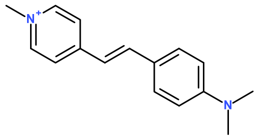<br>CN(C)c1ccc(\C=C\c2cc[n+](C)cc2)cc1	GC VI 17	8	0.903
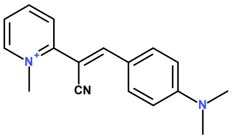<br>CN(C)c1ccc(\C=C(/C#N)\c2cccc[n+]2C)cc1	GC VI 26	100	2
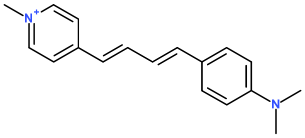<br>CN(C)c1ccc(\C=C\C=C\c2cc[n+](C)cc2)cc1	GC VI 45	16	1.20
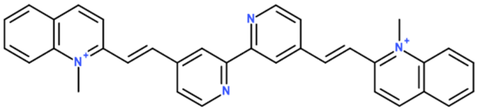<br>C[n+]1c(\C=C\c2ccnc(c2)c3cc(\C=C\c4ccc5ccccc5[n+]4C)ccn3)ccc6ccccc16	GC VI 71	51.2	1.71
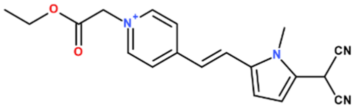<br>CCOC(=O)C[n+]1ccc(\C=C\c2ccc(C(C#N)C#N)n2C)cc1	GC VII 13	100	2
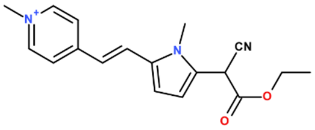<br>CCOC(=O)C(C#N)c1ccc(\C=C\c2cc[n+](C)cc2)n1C	GC VII 16	70	1.85
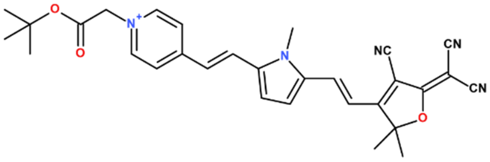<br>Cn1c(\C=C\C2=C(C#N)C(=C(C#N)C#N)OC2(C)C)ccc1\C=C\c3cc[n+]<br>(CC(=O)OC(C)(C)C)cc3	GC VII 41	100	2

Table S2. Continued

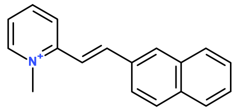 C[n+]1ccccc1\C=C\c2ccc3ccccc3c2	GC VII 46	5.5	0.740
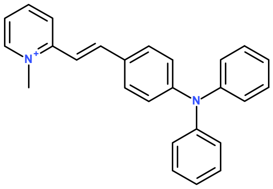 C[n+]1ccccc1\C=C\c2ccc(cc2)N(c3ccccc3)c4ccccc4	GC VII 47	1	0
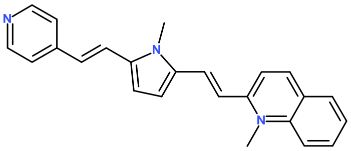 Cn1c(\C=C\C2=CC=C3C=CC=CC3=[N]2C)ccc1\C=C\c4ccncc4	GF I 10	20	1.30
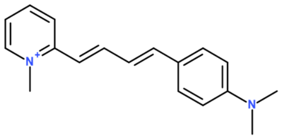 CN(C)c1ccc(\C=C\C=C\c2cccc[n+]2C)cc1	GF I 25	10	1
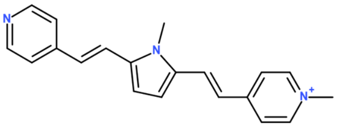 C[n+]1ccc(\C=C\c2ccc(\C=C\c3ccncc3)n2C)cc1	GF I 30	50	1.70
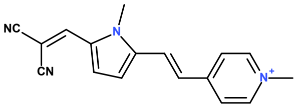 C[n+]1ccc(\C=C\c2ccc(C=C(C#N)C#N)n2C)cc1	GF I 31	100	2
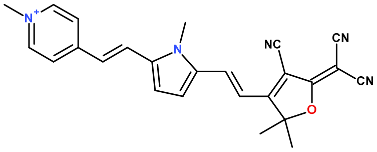 C[n+]1ccc(\C=C\c2ccc(\C=C\C3=C(C#N)C(=C(C#N)C#N)OC3(C)C)n2C)cc1	GF I 32	32	1.51
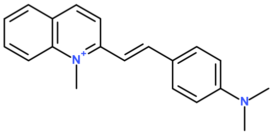 CN(C)c1ccc(\C=C\c2ccc3ccccc3[n+]2C)cc1	PB1	0.35	-0.456

Table S2. Continued

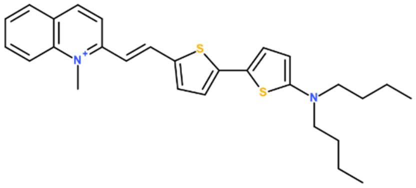<br>CCCCN(CCCC)c1ccc(s1)c2ccc(\C=C\c3ccc4ccccc4[n+]3C)s2	PB2	0.18	-0.745
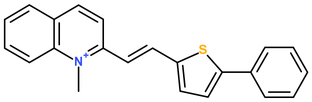<br>C[n+]1c(\C=C\c2ccc(s2)c3ccccc3)ccc4ccccc14	PB3	1.3	0.114
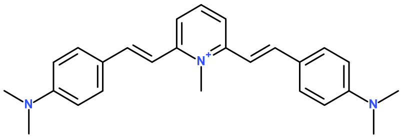<br>CN(C)c1ccc(\C=C\c2cccc(\C=C\c3ccc(cc3)N(C)C)[n+]2C)cc1	PB4	0.33	-0.481
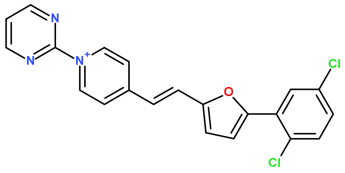<br>Clc1ccc(Cl)c(c1)c2oc(\C=C\c3cc[n+](cc3)c4ncccn4)cc2	PB5	0.95	0.246
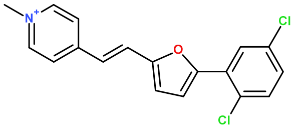<br>C[n+]1ccc(\C=C\c2oc(cc2)c3cc(Cl)ccc3Cl)cc1	PB6	3.22	0.508
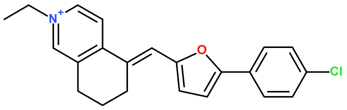<br>CC[n+]1ccc2\C(=C\c3oc(cc3)c4ccc(Cl)cc4)\CCCc2c1	PB7	1.36	0.134
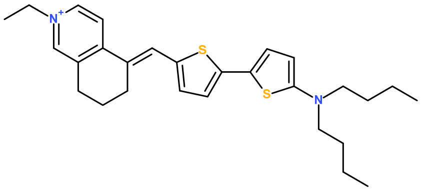<br>CCCCN(CCCC)c1ccc(s1)c2ccc(\C=C\3/CCCc4c[n+](CC)ccc34)s2	PB8	5.04	0.702

Table S2. Continued

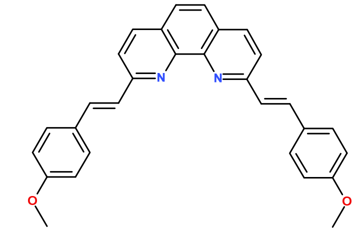 COc1ccc(\C=C\c2ccc3ccc4ccc(\C=C\c5ccc(OC)cc5)nc4c3n2)cc1	PB9	250	2.40
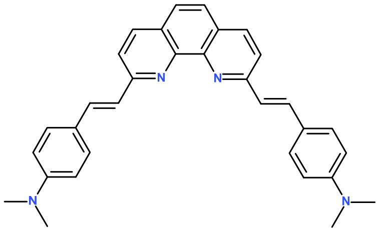 CN(C)c1ccc(\C=C\c2ccc3ccc4ccc(\C=C\c5ccc(cc5)N(C)C)nc4c3n2)cc1	PB10	10.6	1.03
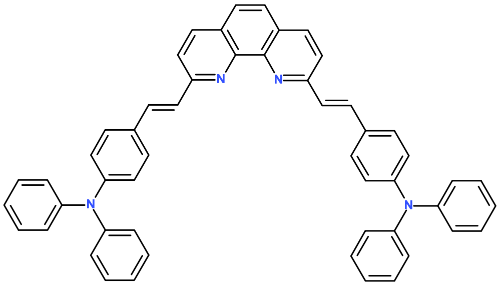 C(=C\c1ccc2ccc3ccc(\C=C\c4ccc(cc4)N(c5ccccc5)c6ccccc6)nc3c2n1)/c7ccc(cc7)N (c8ccccc8)c9ccccc9	PB11	11.5	1.06

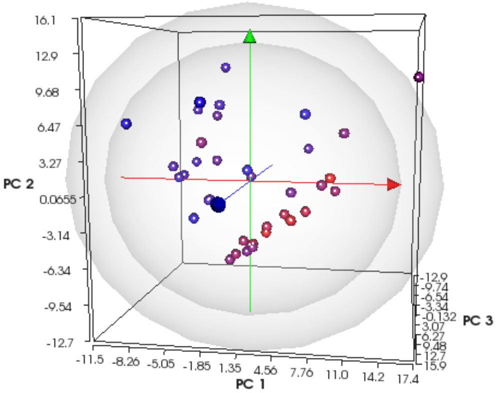

Figure S5. Score plot of Principal Component Analysis (PCA) at the third component (PC1 *vs* PC2 *vs* PC3) for the 38 heteroaromatic compounds tested for cytotoxic activity towards CaCo2 colon-rectal cancer cell line. Compounds are color-coded by their activity values, using a scale from red (actives) to blue (inactives), according to the experimental MIC values.

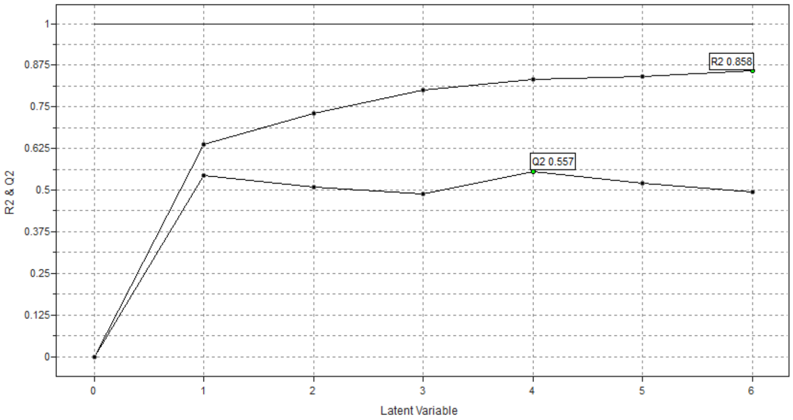

Figure S6. Plot of the coefficient of determination R^2 and cross-validated coefficient of determination (leave-one-out, LOO) Q^2 vs. the number of LVs of the PLS models for cytotoxic activity towards CaCo2 colon-rectal cancer cell line.

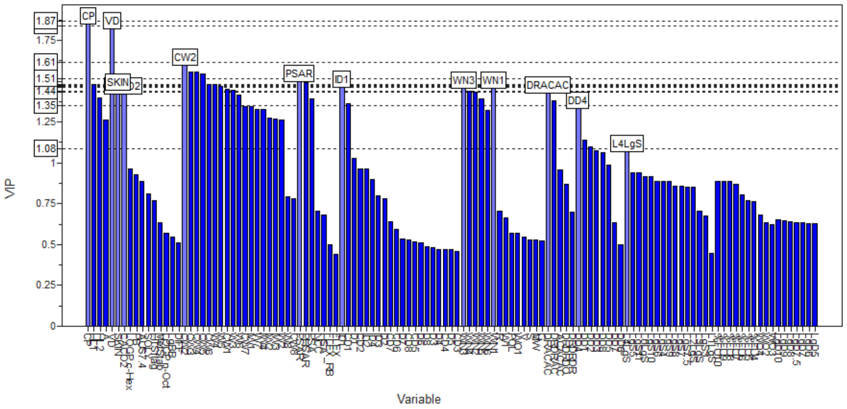

Figure S7. Plot of the Variable Influence on Projection VIP of the PLS models for cytotoxic activity towards CaCo2 colon-rectal cancer cell line.

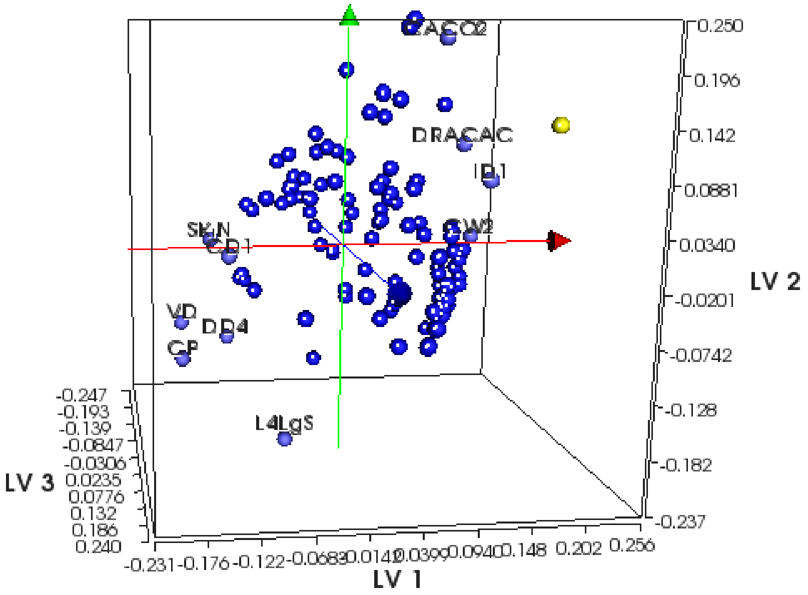

Figure S8. Plot of the Weights for VS+ descriptors at the third latent variable (LV1 *vs* LV2 *vs* LV3) of the PLS models for cytotoxic activity towards CaCo2 colon-rectal cancer cell line. The yellow circle represents the dependent variable the logarithm of the IC_{50}.

Table S3. Dunnett's multiple comparisons test performed for all combinations. * = P-value ≤ 0.05; **= P-value ≤ 0.01; ***=P-value ≤ 0.001; ****= P-value≤ 0.0001.

Dunnett's multiple comparisons test	Mean Diff	95% CI of diff	Below threshold?	Summary	Adjusted P Value
**ANTIBIOTICS 24h**					
Untreated vs. T0	48.44	29.50 to 67.39	Yes	****	<0.0001
Untreated vs. Linezolid MIC	-3.090	-36.67 to 30.49	No	ns	0.9997
Untreated vs. Linezolid subMIC	7.008	-26.57 to 40.59	No	ns	0.9993
Untreated vs. Gentamicin MIC	-7.797	-41.38 to 25.78	No	ns	0.9992
Untreated vs. Gentamicin subMIC	18.12	-15.46 to 51.70	No	ns	0.7346
Untreated vs. Ampicillin MIC	2.723	-30.86 to 36.30	No	ns	0.9998
Untreated vs. Ampicillin subMIC	-5.167	-38.75 to 28.41	No	ns	0.9996
Untreated vs. Erythromycin MIC	2.213	-31.37 to 35.79	No	ns	0.9998
Untreated vs. Erythromycin subMIC	-9.070	-42.65 to 24.51	No	ns	0.9963
Untreated vs. Rifampicin MIC	16.68	-16.90 to 50.26	No	ns	0.8188
Untreated vs. Rifampicin subMIC	3.783	-29.80 to 37.36	No	ns	0.9997
**ANTIBIOTICS + PB4 0.2 μM 24h**					
Untreated vs. T0	52.99	37.65 to 68.32	Yes	****	<0.0001
Untreated vs. Linezolid MIC	10.99	-16.19 to 38.17	No	ns	0.9435
Untreated vs. Linezolid subMIC	22.91	-4.265 to 50.09	No	ns	0.1618
Untreated vs. Gentamicin MIC	29.40	2.225 to 56.58	Yes	*	0.0252
Untreated vs. Gentamicin subMIC	22.53	-4.648 to 49.71	No	ns	0.1778
Untreated vs. Ampicillin MIC	21.13	-6.045 to 48.31	No	ns	0.2471
Untreated vs. Ampicillin subMIC	19.39	-7.785 to 46.57	No	ns	0.3570
Untreated vs. Erythromycin MIC	21.39	-5.793 to 48.56	No	ns	0.2332
Untreated vs. Erythromycin subMIC	30.08	2.905 to 57.26	Yes	*	0.0202
Untreated vs. Rifampicin MIC	28.09	0.9099 to 55.27	Yes	*	0.0380
Untreated vs. Rifampicin subMIC	24.31	-2.865 to 51.49	No	ns	0.1126
**ANTIBIOTICS 48h**					
Untreated vs. T0	136.6	104.1 to 169.1	Yes	****	<0.0001
Untreated vs. Linezolid MIC	-14.37	-71.94 to 43.20	No	ns	0.9968
Untreated vs. Linezolid subMIC	-9.069	-66.64 to 48.50	No	ns	0.9995
Untreated vs. Gentamicin MIC	-8.939	-66.51 to 48.63	No	ns	0.9995
Untreated vs. Gentamicin subMIC	-33.12	-90.69 to 24.45	No	ns	0.6570
Untreated vs. Ampicillin MIC	-82.54	-140.1 to -24.97	Yes	***	0.0008
Untreated vs. Ampicillin subMIC	-8.984	-66.55 to 48.58	No	ns	0.9995
Untreated vs. Erythromycin MIC	-30.40	-87.97 to 27.16	No	ns	0.7585
Untreated vs. Erythromycin subMIC	-42.07	-99.64 to 15.50	No	ns	0.3247
Untreated vs. Rifampicin MIC	-44.02	-101.6 to 13.54	No	ns	0.2669
Untreated vs. Rifampicin subMIC	-27.05	-84.62 to 30.51	No	ns	0.8635
**ANTIBIOTICS + PB4 0.2 μM 48h**					

Untreated vs. T0	84.25	63.16 to 105.3	Yes	****	<0.0001
Untreated vs. Linezolid MIC	40.56	3.180 to 77.95	Yes	*	0.0245
Untreated vs. Linezolid subMIC	55.66	18.28 to 93.05	Yes	***	0.0005
Untreated vs. Gentamicin MIC	41.74	4.358 to 79.13	Yes	*	0.0186
Untreated vs. Gentamicin subMIC	86.73	49.35 to 124.1	Yes	****	<0.0001
Untreated vs. Ampicillin MIC	58.50	21.12 to 95.89	Yes	***	0.0002
Untreated vs. Ampicillin subMIC	49.22	11.83 to 86.60	Yes	**	0.0028
Untreated vs. Erythromycin MIC	70.36	32.97 to 107.7	Yes	****	<0.0001
Untreated vs. Erythromycin subMIC	30.43	-6.955 to 67.81	No	ns	0.1963
Untreated vs. Rifampicin MIC	27.48	-9.900 to 64.87	No	ns	0.3169
Untreated vs. Rifampicin subMIC	22.49	-14.89 to 59.88	No	ns	0.5974

Table S4. Values obtained from the MTT assay normalised with respect to T0 with their standard deviations.
AA = arithmetic average ST.DEV.= Standard Deviation.

	ANTIBIOTICS 24H									
	UNTREATED									
	127.3	87.0	147.8	163.7	135.4	148.6	151.9	225.0	137.3	157.8
	143.2	46.2	138.5	142.2	210.1	161.7	193.9	154.9	125.6	156.3
	153.7	153.9	150.7	167.3	158.6	160.2	173.2	198.2	127.4	134.0
	120.6	171.7	114.7	130.0	143.2	123.4	134.4	135.1	156.8	133.7
AA	147.4									
ST.DEV.	30.6									
	LIN MIC	LIN subMIC	GEN MIC	GEN subMIC	AMP MIC	AMP subMIC	ERI MIC	ERI subMIC	RIF MIC	RIF subMIC
	157.5	133.4	173.7	93.3	144.7	163.1	156.1	209.7	121.5	149.3
	138.3	142.5	162.7	138.3	152.7	141.2	163.4	151.2	144.4	150.0
	161.7	137.3	136.4	118.4	141.0	169.7	129.0	118.8	112.7	139.1
	156.8	160.7	160.2	179.3	152.5	148.6	144.6	158.5	156.6	148.3
	LIN MIC	LIN subMIC	GEN MIC	GEN subMIC	AMP MIC	AMP subMIC	ERI MIC	ERI subMIC	RIF MIC	RIF subMIC
AA	153.6	143.5	158.3	132.3	147.7	155.6	148.3	159.5	133.8	146.7
ST.DEV.	10.4	12.1	15.7	36.3	5.8	13.0	15.0	37.6	20.3	5.1

	ANTIBIOTICS 48H									
	UNTREATED									
	186.5	210.6	226.7	234.7	238.4	237.0	273.3	249.8	272.0	191.0
	242.6	273.2	208.5	169.8	240.3	443.9	205.1	290.5	245.0	241.4
	210.2	268.6	169.8	188.7	215.7	430.6	343.2	319.7	236.9	257.4
	227.7	304.6	249.4	221.3	206.3	271.8	224.6	269.4	223.1	232.8
AA	248.8									
ST.DEV.	57.7									
	LIN MIC	LIN subMIC	GEN MIC	GEN subMIC	AMP MIC	AMP subMIC	ERI MIC	ERI subMIC	RIF MIC	RIF subMIC
	182.1	252.1	221.1	233.3	399.9	273.3	316.1	249.4	358.2	274.7
	264.2	283.5	224.6	275.4	275.7	237.4	253.0	270.8	269.1	236.0
	256.7	239.4	250.9	222.6	327.1	206.3	296.9	325.1	281.1	293.7
	309.0	215.7	293.5	355.6	281.8	273.3	210.1	277.4	222.1	258.2
	LIN MIC	LIN subMIC	GEN MIC	GEN subMIC	AMP MIC	AMP subMIC	ERI MIC	ERI subMIC	RIF MIC	RIF subMIC
AA	253.0	247.7	247.6	271.7	321.1	247.6	269.0	280.7	282.6	265.7
ST.DEV.	52.6	28.3	33.4	60.4	57.3	32.3	47.4	31.9	56.4	24.5

ANTIBIOTICS and PB4 0.2 uM 24H									
UNTREATED									
142.5	174.3	161.0	150.2	135.6	164.1	145.1	153.7	155.6	162.7
130.0	177.0	222.3	140.1	153.7	138.5	176.5	170.2	150.5	151.0
130.6	193.9	149.5	175.4	146.9	162.2	170.2	189.2	132.2	160.3
141.7	151.9	122.0	123.9	149.7	138.3	161.5	166.1	146.6	133.7

ANTIBIOTICS and PB4 0.2 uM 48H									
UNTREATED									
225.2	198.3	212.4	180.0	253.0	191.0	193.3	218.2	174.4	170.0
264.2	177.0	186.8	146.4	186.6	178.7	140.3	192.1	197.8	157.5
172.0	162.9	223.5	191.4	153.7	226.5	180.5	204.5	160.7	199.4
199.2	214.5	204.1	144.2	139.0	214.5	142.2	150.8	164.4	159.7

AA	155.0									
ST.DEV.	20.2									
	LIN	LIN	GEN	GEN	AMP	AMP	ERI	ERI	RIF	RIF
	MIC	subMIC	MIC	subMIC	MIC	subMIC	MIC	subMIC	MIC	subMIC
	149.0	118.9	131.8	129.6	144.9	154.7	129.8	140.1	100.3	136.1
	130.6	143.2	110.1	126.4	149.3	119.1	130.0	123.0	140.1	131.0
	144.6	119.3	129.0	115.4	111.1	136.1	133.5	114.4	128.3	134.0
	151.9	146.9	131.5	158.5	130.1	132.5	141.2	122.2	139.0	121.7
	LIN	LIN	GEN	GEN	AMP	AMP	ERI	ERI	RIF	RIF
	MIC	subMIC	MIC	subMIC	MIC	subMIC	MIC	subMIC	MIC	subMIC
AA	144.0	132.1	125.6	132.5	133.9	135.6	133.6	124.9	126.9	130.7
ST.DEV.	9.4	15.1	10.4	18.4	17.2	14.7	5.3	10.9	18.5	6.4

AA	186.3									
ST.DEV.	30.3									
	LIN	LIN	GEN	GEN	AMP	AMP	ERI	ERI	RIF	RIF
	MIC	subMIC	MIC	subMIC	MIC	subMIC	MIC	subMIC	MIC	subMIC
	157.3	150.7			146.9	138.5		134.4	134.7	160.5
	143.0	120.1	164.8	91.8	152.0	130.8	105.9	178.2	164.9	193.8
	145.2	136.8	123.7	91.3	99.9	152.2	121.8	179.2	177.1	139.3
	137.3	114.9	134.9	114.7	112.2	126.7	124.7	131.7		161.5
	LIN	LIN	GEN	GEN	AMP	AMP	ERI	ERI	RIF	RIF
	MIC	subMIC	MIC	subMIC	MIC	subMIC	MIC	subMIC	MIC	subMIC
AA	145.7	130.6	141.1	99.3	127.8	137.1	117.5	155.8	158.9	163.8
ST.DEV.	8.4	16.3	21.2	13.4	25.7	11.2	10.1	26.4	21.8	22.5
